# Interpretation According to Clone-Specific PD-L1 Cutoffs Reveals Better Concordance in Muscle-Invasive Urothelial Carcinoma

**DOI:** 10.3390/diagnostics11030448

**Published:** 2021-03-05

**Authors:** Tzu-Hao Huang, Wei Cheng, Yeh-Han Wang

**Affiliations:** 1 Department of Urology, Taipei Veterans General Hospital, Taipei 11217, Taiwan; jayhuangx@gmail.com; 2 School of Medicine, National Yang-Ming University, Taipei 11221, Taiwan; 3 Department of Anatomic Pathology, Keelung Hospital, Ministry of Health and Welfare, Keelung 20141, Taiwan; kln8301@kln.mohw.gov.tw; 4 College of Nursing, National Taipei University of Nursing and Health Sciences, Taipei 11219, Taiwan; 5 Department of Nursing, Ching Kuo Institute of Management and Health, Keelung 20301, Taiwan; 6 Department of Pathology, Heping Fuyou Branch, Taipei City Hospital, Taipei 10065, Taiwan; 7 Institute of Public Health, National Yang-Ming University, Taipei 11221, Taiwan

**Keywords:** PD-L1, scoring algorithm, harmonization, urothelial carcinoma, concordance, molecular phenotype

## Abstract

Because immune checkpoint inhibitors have been approved for treating advanced urothelial carcinoma (UC), programmed death-ligand 1 (PD-L1) immunohistochemistry (IHC) assays have been widely used as companion or complementary diagnostic tests for predicting treatment outcomes. Because different clones, scoring algorithms, and cutoffs have been used for interpretation, this study investigated the variation, correlation, and concordance of four validated PD-L1 clones (SP142, SP263, 22C3, and 28-8) and proposed a practical solution for the harmonization of PD-L1 IHC. A tissue microarray, including 46 muscle-invasive UCs, was constructed for PD-L1 testing with the four clones. Tumor cell (TC) and immune cell (IC) expression was analyzed. SP142 had significantly low TC expression, whereas SP263, 22C3, and 28-8 exhibited a moderate correlation (rho ≥ 0.6), with almost perfect concordance (intraclass correlation coefficient > 0.8) in TC expression. Fair to moderate correlation and concordance were observed in IC expression in most pairwise comparisons of clones. Substantial concordance (kappa > 0.6) was noted when high PD-L1 expression was defined by applying clone-specific cutoffs to each clone. Our findings imply that a universal cutoff value is not feasible for UC; we propose that PD-L1 IHC assays for UC should be interpreted according to a clone-specific scoring algorithm and cutoff value.

## 1. Introduction

Because several immune checkpoint inhibitors (ICIs) have been approved as the first- and second-line treatment for advanced urothelial carcinoma (UC), various molecular tests have been proposed as predictive biomarkers, such as tumor mutation burden, programmed death-ligand 1 (PD-L1) immunohistochemistry (IHC) assays, and The Cancer Genome Atlas phenotype [1,2]. Among them, PD-L1 IHC is most widely employed in the pathology laboratory setting. High PD-L1 expression has been demonstrated to correlate with a better ICI treatment response, and the expression is associated with some histomorphological features, such as squamous differentiation and the basal phenotype of UC [3,4,5,6]. To date, four commercialized PD-L1 clones (Dako 22C3 PharmDx, 28-8 PharmDx, Ventana SP142, and SP263) have been approved as companion or complementary diagnostics by the US Food and Drug Administration (FDA) for determining high PD-L1 expression in patients with UC [7,8,9].

Although PD-L1 IHC has been widely used in diagnosis, its standardization has been challenging; various scoring algorithms and cutoff values have been applied across PD-L1 clones and cancer types. The harmonization of PD-L1 IHC has thus been identified as an essential issue in clinical practice. Many studies have investigated the degree of concordance and interchangeability between clones. The results have been conflicting, with varying findings across cancer types. The first attempt to harmonize PD-L1 staining was the blueprint study for non-small-cell lung cancer (NSCLC) [10,11]. In a phase 1 blueprint study, researchers concluded that the tumor cell (TC) expression between SP263, 22C3, and 28-8 was comparable, that SP142 had a lower expression in TCs, and that the immune cell (IC) expression was incomparable between clones [10]. The study also suggested a harmonization strategy; with a similar TC scoring algorithm and cutoff, PD-L1 clones can be interchangeable. However, in NSCLC most clinical trials defined PD-L1 status on the basis of TC expression [12]. Only trials of atezolizumab have employed SP142 staining on either TCs or ICs as the criterion for determining high PD-L1 expression [13]. Such results cannot be applied to UC because the definitions of high PD-L1 expression proposed in trials of ICIs in UC were considerably different between clones. 

For UC, the cutoffs and scoring algorithms for low/high PD-L1 expression were different, with between-clone variation in the target cells included for counting [14]. For example, only IC staining was measured in the scoring algorithm of SP142, whereas the high expression of clone 28-8 was solely defined by its TC expression. In clone 22C3, a unique scoring system, the combined proportion score (CPS), was employed for including both TC and IC expression; however, >25% expression of either TCs or ICs was reported as positive in SP263. In addition, the target cells included for scoring were different. Table 1 summarizes the details of scoring protocols, definitions of high PD-L1 expression in UC, and target cells for scoring. This study evaluated the variation in scoring TC and IC expression between clones and the concordance of high PD-L1 expression defined by each clone to determine whether a better harmonization strategy can be applied for UC.

## 2. Materials and Methods 

### 2.1. Tissue Microarray Construction and PD-L1 IHC Staining 

This study was approved by the Institutional Review Board of Taipei Veterans General Hospital (TVGH) (2020-03-007CC, 12 March 2020). Forty-six tissue blocks of muscle-invasive UC obtained during 2016–2018 were retrieved from the biobank of TVGH. Specimens obtained from radical cystectomy or transurethral resection of bladder tumor were accepted. After pathology review, for each specimen one or two 2 mm cores were selected from invasive areas, depending on the size of the area, for constructing a tissue microarray (TMA). 

Next, four FDA-approved PD-L1 clones were applied in IHC staining: Ventana SP142 and Ventana SP263 (Ventana Medical Systems, Tucson, AZ, USA), and 22c3 pharmDx and 28-8 pharmDx (Dako Agilent Technologies, Santa Clara, CA, USA). The PD-L1 staining was processed on two platforms—the Benchmark Ultra System (SP142 and SP263) and Autostainer Link 48 (22C3 and 28-8)—as per the manufacturers’ instructions.

The correlation between high PD-L1 expression and UC phenotype was also investigated. IHC stains of GATA-3 (ZETA L50-823, 1:100) and cytokeratin (CK) 5/6 (RM341, 1:1000) were applied. On the basis of the pattern of coexpression of GATA-3 and CK 5/6 proposed by Guo et al., three subgroups were defined: basal type (negative GATA-3 and diffuse positive CK5/6), luminal type (positive GATA-3 and negative/focal positive CK5/6), and undetermined (double negative or double positive) [15].

### 2.2. Interpretation of PD-L1 Expression

The interpretation of PD-L1 expression included both TC and IC staining. The scoring was performed by a licensed anatomic pathologist (Y.W.) in a blinded fashion and followed the approved interpretation guidance published by the two manufacturers. For TC and IC scoring algorithms not included in the interpretation for UC, a scoring algorithm approved for other cancer types was employed. For SP142, the guidance proposed for scoring TC expression in NSCLC was applied to UC cases. However, the IC scoring of 22C3 was measured on the basis of the CPS with a further exclusion of its TC scoring. We applied the same algorithm to 28-8 for measuring IC expression because the two clones were produced by the same manufacturer. TC and IC scores were recorded separately by case. For those with two cores in the TMA, the average TC and IC scores were calculated.

Using the cutoffs validated by the four clones (Table 1), cases with a high PD-L1 expression (positive cases) were thus individually defined in accordance with a paired clone and its cutoff. To evaluate whether the cutoff proposed for one clone could be applied to other PD-L1 clones, we used all four cutoffs to define low/high PD-L1 expression for each clone.

### 2.3. Statistical Analysis

Data analysis was performed using SPSS (version 25; IBM, Armonk, NY, USA). Variations in PD-L1 TC and IC scores were analyzed using a paired-sample t test. Spearman’s rank correlation coefficient (rho) was calculated for evaluating the correlation between PD-L1 clones. To describe the strength of correlation, we used the definition proposed by Chan: a rho value from >0 to <0.3 was considered to indicate weak correlation, 0.3 to <0.6 to indicate fair correlation, 0.6 to <0.8 to indicate moderate correlation, and 0.8 to <1 to indicate strong correlation [16,17]. The concordance of TC and IC scores and the positivity determined using clone-specific cutoffs were assessed using the intraclass correlation coefficient (ICC) for continuous variables and Fleiss’ or Cohen’s kappa for categorical variables (depending on the number of raters). Both the overall and pairwise concordance was analyzed. An ICC or kappa value of 0–0.2 was considered to indicate slight concordance, 0.21–0.4 to indicate fair concordance, 0.41–0.6 to indicate moderate concordance, 0.61–0.8 to indicate substantial concordance, and 0.81–1 to indicate almost perfect concordance [18]. Fisher’s exact test was performed to evaluate the association between high PD-L1 expression and UC phenotypes.

## 3. Results

**Figure 1:**
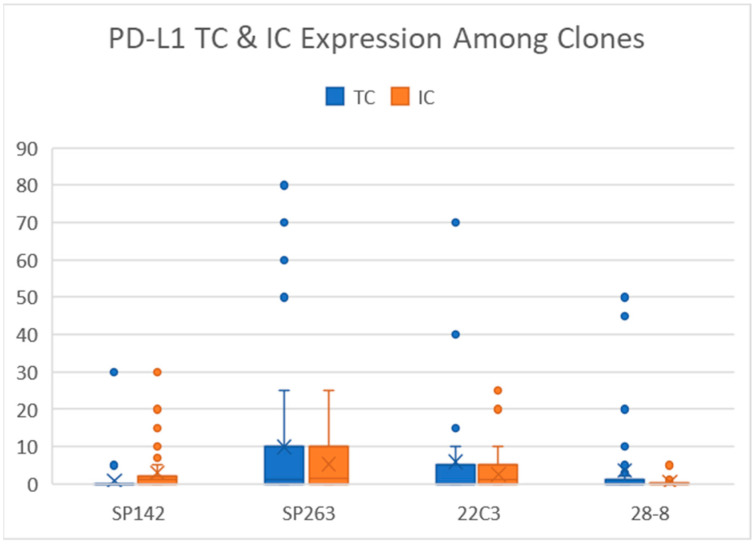
TC and IC scores of the four PD-L1 clones. Overall, SP263 had the highest expression of TCs and ICs. SP142 had negligible TC expression, and 28-8 had extremely low IC expression.

Among the 46 specimens of muscle-invasive UC, the average PD-L1 TC expression ranged from 0.87% to 9.89% (SP142: 0.87%, SP263: 9.89%, 22C3: 5.93%, and 28-8: 3.61%) and the average IC expression ranged from 0.57% to 5.30% (SP142: 2.98%, SP263: 5.30%, 22C3: 2.59%, and 28-8: 0.57%). Among the four PD-L1 clones, SP263 had the highest expression for both TCs and ICs; the lowest TC and IC expression was observed in SP142 and 28-8, respectively. Figure 1 presents the distribution of the TC and IC scores of each clone. In a pairwise comparison using the paired t test, all the TC expression exhibited significant differences between any two clones. As for the IC expression, no significant difference in numerical scores were detected between SP142 and SP263 (*p* = 0.08) and between SP142 and 22C3 and (*p* = 0.69).

### 3.1. Correlation of TC and IC Expression between Clones

**Figure 2:**
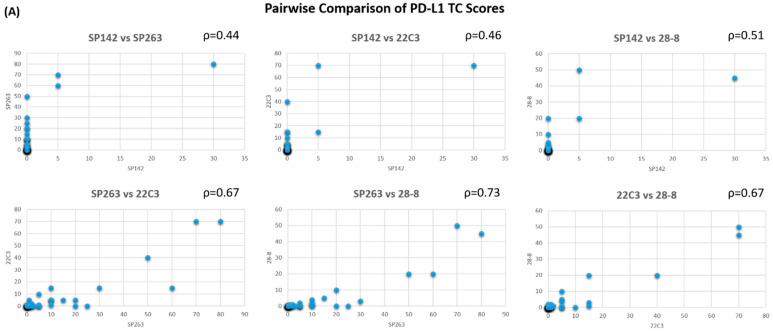

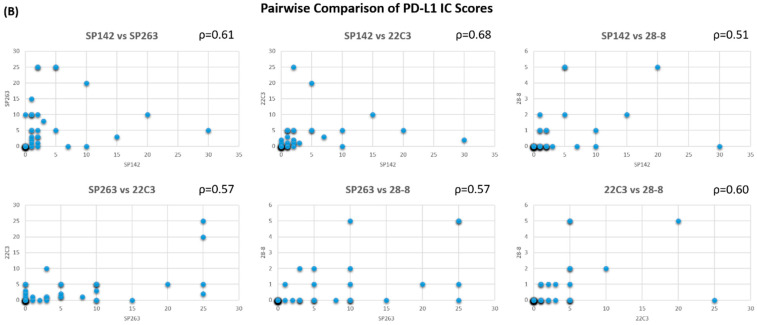
Pairwise comparison and correlation of (**A**) TC scores and (**B**) IC scores between clones, with the Spearman’s rank correlation coefficient in the upper right corner of each plot.

For TC scores, SP142 had only fair correlations with SP263, 22C3, and 28-8 (rho = 0.44, 0.46, and 0.51, respectively), whereas SP263, 22C3, and 28-8 had moderate correlations with each other. Compared to that for the TC scores, the pairwise comparison of IC scores by clones revealed a similar strength of correlation. All the Spearman’s correlation coefficients ranged from 0.51 to 0.68, indicating fair to moderate correlations between any two clones. Figure 2 illustrates the pairwise comparison of PD-L1 TC and IC expression by clones.

### 3.2. Concordance of PD-L1 Expression between Clones

Although the TC expression was moderately correlated among PD-L1 clones except for SP142, the concordance of PD-L1 expression was almost perfect among SP263, 22C3, and 28-8 (ICC for SP263 vs. 22C3: 0.92, SP263 vs. 28-8: 0.84, and 22C3 vs. 28-2: 0.93). Nearly all the IC expression between clones had only fair concordance (ICC: 0.21–0.4), except for that between SP263 and 22C3. The ICC for SP263 and 22C3 expression on ICs was 0.69, indicating substantial concordance. Detailed ICC data are listed in Table 2.

Fourteen (30%) cases were positive for at least one PD-L1 clone when considering the clone-specific cutoff, with the positive rate ranging from 13.0% to 26.1% by clone (SP142: 19.6%, SP263: 19.6%, 22C3: 26.1%, and 28-8: 13.0%); only five (10.9%) cases were consistently positive for all PD-L1 clones. Substantial agreement was observed in the overall concordance of high PD-L1 expression (Fleiss kappa = 0.678, 95% confidence interval (CI): 0.56–0.8). Pairwise concordance revealed a similar degree of agreement with Cohen’s kappa values (0.60–0.77; Table 2).

**Figure 3:**
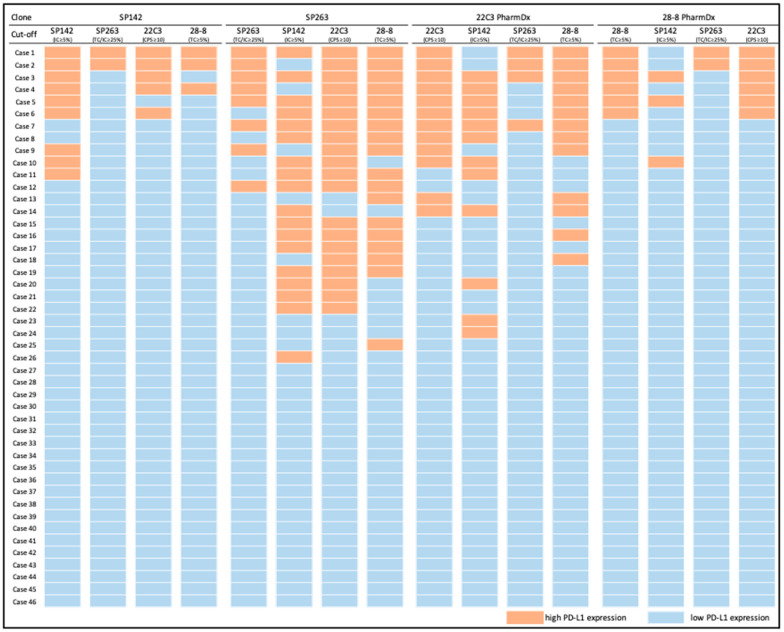
Low/high PD-L1 expression defined by a combination of staining clone and four clone-specific cutoffs.

When a universal cutoff was applied, the concordance was lower (Fleiss’s kappa for applying SP142 cutoff: 0.32 (CI: 0.20–0.44), SP263 cutoff: 0.50 (CI: 0.39–0.62), 22C3 cutoff: 0.44 (CI: 0.33–0.56), and 28-8 cutoff: 0.43 (CI: 0.31–0.54)). The SP142 cutoff considered only IC expression, resulting in a considerable overinterpretation of PD-L1 expression for SP263 and 22C3 and underinterpretation for 28-8. When the SP263 cutoff was applied, which considered both TCs and ICs, the positive rate was considerably lower for the other clones because they all had a lower expression than SP263. The detailed results are illustrated in Figure 3.

### 3.3. Association of High PD-L1 Expression between Phenotypes

**Figure 4:**
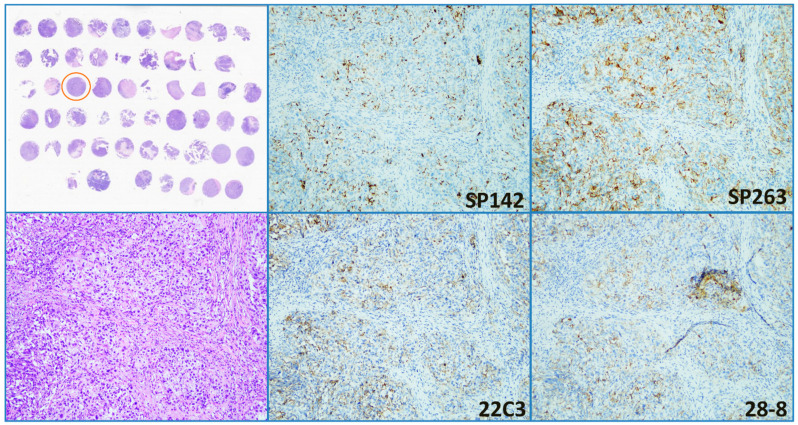
High PD-L1 expression demonstrated by the four clones in a case (marked by a circle on the TMA map).

On the basis of the expression of GATA-3 and CK 5/6, 36 (78.2%) of 46 cases were concluded to be of the luminal type, 5 (10.9%) the basal type, and 5 (10.9%) the undetermined type (3 with double-positive staining and 2 with double-negative staining). Basal-type UC had the highest PD-L1 positive rate (3/5, 60%), and three cases consistently expressed high PD-L1 for all four clones (Figure 4). By contrast, the luminal-type UC had variable PD-L1 expression between clones; only one case was positive for all four clones. Table 3 presents the distribution of cases with low and high PD-L1 expression among phenotypes. No association had significant difference between phenotypes and SP142, SP263, and 22C3 clones. 

## 4. Discussion

Various studies have attempted to harmonize PD-L1 IHC assays in different cancer types, primarily in NSCLC [10,11,19]. Although some head-to-head comparisons have yielded mixed results, low TC expression has been consistently observed in SP142 compared with the other three clones [10,20,21,22], which agrees with our results; this explains the merely fair correlation with other clones. Thus, applying any TC scoring algorithm and cutoffs to SP142 for assessing PD-L1 expression would be inappropriate. In addition, even though SP263, 22C3, and 28-8 have better (moderate) correlation and there is almost perfect concordance between their TC scores, the difference in their numeric values, especially in the TC scores of SP263 and 22C3 or 28-8, makes it difficult to specify a single TC cutoff for determining low/high PD-L1 expression across clones. In other words, the harmonization strategy for NSCLC is not applicable for UC.

The comparison of IC expression across clones is more complex. Although a blueprint study revealed incomparable PD-L1 expression on ICs [10], Zajac et al. demonstrated a strong correlation (rho > 0.8) of IC scores if the same algorithm was employed [21]. Our data indicated only fair correlations between the clones, and most pairwise comparisons also revealed only fair concordance in scores. Considering that three algorithms with different denominators were used, it was nearly impossible to implement a unified IC scoring algorithm and cutoff for defining positivity.

Approximately 20% of cases of UC have high PD-L1 expression [22], which is consistent with our results, although the rate of 28-8 was slightly lower. Despite the substantial between-clone variation in TC and IC scores, the high PD-L1 expression of each clone exhibited a moderate to good concordance when clone-specific cutoffs were applied. Moreover, based on the TC and IC expression, SP142 and 28-8 seemed to be situated at the two extremes of the PD-L1 expression spectrum. SP142 had the lowest TC expression, whereas 28-8 had the lowest IC expression. A scoring algorithm that excluded TC and IC expression, respectively, was used for them. The application of a universal cutoff—using TC, IC, or even a mixed score—to determine the positivity of PD-L1 expression therefore led to considerable over- and under-interpretation (Figure 3). Accordingly, we propose a new strategy to harmonize PD-L1 IHC in UC cases: a slide stained by one clone should be interpreted according to the clone-specific scoring algorithm and cutoff value.

Basal-type UC has been shown to correlate with poor prognosis and high PD-L1 expression [6]. Our study obtained similar results, although the number of cases was not sufficient. Basal-type UC cases not only have the highest positive rate for PD-L1 expression but also demonstrate a 100% agreement of high PD-L1 expression across the four clones. By contrast, more variable results are observed in the luminal type. In our study, 28-8 had the least expression in luminal-type UC, whereas 22C3 was the most sensitive clone that defined most luminal-type UC cases with high PD-L1 expression. However, clinical validation by treatment outcomes is warranted to establish whether the variation resulted from tumor nature or the performance of PD-L1 clones. Further clinical validation studies should be conducted to investigate which clone can best predict the treatment outcome of ICIs in patients with luminal-type UC.

Any study using a TMA has limitations in that the results can be biased by tumor heterogeneity. Tretiacova et al. investigated the concordance between primary and metastatic bladder carcinomas and reported moderate to high concordance between them and low intratumor heterogeneity of PD-L1 expression [23]. To minimize the bias caused by tumor heterogeneity, we selected only deep invasive areas to construct the TMA, and up to two cores were extracted if there was a sufficient area of invasion. Another limitation is the small number of cases included in the study, thus limiting the statistical differential power.

## 5. Conclusions

In summary, our study not only provides evidence to support the findings of other studies regarding PD-L1 comparison and harmonization across clones but also proposes a practical solution for harmonizing PD-L1 testing results in UC cases. Because the numeric results of PD-L1 TC and IC expression may be more discrepant in UC cases, a better concordance between clones is achieved when clone-specific cutoffs are used. Further research on the clinical validation of clone-specific cutoffs and scoring algorithms is needed to guide clinical decision-making on the basis of the more precise use of PD-L1 IHC for patients with UC, especially luminal-type UC.

## Figures and Tables

**Table 1:** Scoring algorithm and definition of immune cells among PD-L1 clones.

Clone	Scoring Algorithm	Definition of IC Included for Scoring	Cut-Offs
SP142	$IC\left(\%\right)=\frac{Areas\;occupied\;by\;positive\;IC}{Tumor\;area}$	All immune cells in the intratumoral and contiguous peritumoral stroma.	IC ≥ 5%
SP263	$TC\left(\%\right)=\frac{Positive\;TC\;number}{Total\;viable\;TC\;number}$ or $IC\left(\%\right)=\frac{Areas\;occupied\;by\;positive\;IC}{Tumor\;area\;occupied\;by\;immune\;cells}$	All immune cells within the tumor reactive stroma, between the tumor islands and those invading the tumor proper.	TC ≥ 25% or IC ≥ 25%
22C3	$CPS=\frac{Positive\;TC\;number+positive\;IC\;number}{Total\;viable\;TC\;number}$	Only mononuclear inflammatory cells, including lymphocytes, macrophages/ histiocytes.	CPS ≥ 10
28-8	$TC\left(\%\right)=\frac{Positive\;TC\;number}{Total\;viable\;TC\;number}$	NA	TC ≥ 5%

CPS: combined positive score, IC: immune cell, NA: not applicable, TC: tumor cell.

**Table 2:** Concordance of PD-L1 Expression Between PD-L1 Clones.

PD-L1 Clones	SP142	SP263	22C3	28-8
SP142	1	TC: 0.41 (−0.1–0.67) IC: 0.29 (−0.24–0.60)	TC: 0.53 (0.17–0.74) IC: 0.36 (−0.17–0.65)	TC: 0.68 (0.48–0.82) IC: 0.25 (−0.24–0.57)
SP263	0.72 (0.47–0.98)	1	TC: 0.92 (0.83–0.96) IC: 0.69 (0.41–0.83)	TC: 0.84 (0.59–0.92) IC: 0.25 (−0.19–0.55)
22C3	0.69 (0.45–0.94)	0.69 (0.45–0.94)	1	TC: 0.93 (0.87–0.97) IC: 0.34 (−0.12–0.62)
28-8	0.76 (0.51–1.01)	0.61 (0.30–91)	0.60 (0.32–0.87)	1

The intraclass correlation coefficient (ICC) with 95% confidence interval for scoring concordance of PD-L1 TC and IC expression between clones is listed in the right upper part. Cohen’s kappa for concordance of positivity is listed in the left lower part.

**Table 3:** Distribution of high PD-L1 expression cases among phenotypes.

Clone	SP142 (*p* = 0.05)		SP263 (*p* = 0.05)		22C3 (*p* = 0.07)		28-8 (*p* = 0.01)	
Expression	Low	High	Low	High	Low	High	Low	High
Luminal type	31	5	31	5	29	7	34	2
Basal type	2	3	2	3	2	3	2	3
Undetermined	4	1	4	1	3	2	4	1
Total	37	9	37	9	34	12	40	6

## Data Availability

Data sharing not applicable. No new data were created or analyzed in this study. Data sharing is not applicable to this article.
